# Effects of Operating Parameters on Measurements of Biochemical Oxygen Demand Using a Mediatorless Microbial Fuel Cell Biosensor

**DOI:** 10.3390/s16010035

**Published:** 2015-12-28

**Authors:** Min-Chi Hsieh, Chiu-Yu Cheng, Man-Hai Liu, Ying-Chien Chung

**Affiliations:** 1 Department of Biological Science and Technology, China University of Science and Technology, Taipei 11581, Taiwan; mickey-2068@yahoo.com.tw; 2 Department of Food Science, China University of Science and Technology, Taipei 11581, Taiwan; manhailiu@cc.cust.edu.tw

**Keywords:** biochemical oxygen demand, biosensor, microbial fuel cell, wastewater

## Abstract

The conventional Biochemical Oxygen Demand (BOD) method takes five days to analyze samples. A microbial fuel cell (MFC) may be an alternate tool for rapid BOD determination in water. However, a MFC biosensor for continuous BOD measurements of water samples is still unavailable. In this study, a MFC biosensor inoculated with known mixed cultures was used to determine the BOD concentration. Effects of important parameters on establishing a calibration curve between the BOD concentration and output signal from the MFC were evaluated. The results indicate monosaccharides were good fuel, and methionine, phenylalanine, and ethanol were poor fuels for electricity generation by the MFC. Ions in the influent did not significantly affect the MFC performance. CN^−^ in the influent could alleviate the effect of antagonistic electron acceptors on the MFC performance. The regression equation for BOD concentration and current density of the biosensor was *y* = 0.0145*x* + 0.3317. It was adopted to measure accurately and continuously the BOD concentration in actual water samples at an acceptable error margin. These results clearly show the developed MFC biosensor has great potential as an alternative BOD sensing device for online measurements of wastewater BOD.

## 1. Introduction

BOD is an international regulatory environment index for monitoring biodegradable organic pollutants in water. In industries, continuous monitoring of organic loads is very important in order to comply with regulatory requirements. Thus, a rapid method for online or *in situ* analysis of organic loads is a desirable option, as opposed to a five-day test using the conventional BOD method [1]. Since the conventional test is time-consuming and requires extensive training to achieve reproducible results, it is not suitable for active intervention, environmental monitoring, or process control [1]. Some alternative techniques have been developed to overcome the disadvantages of the conventional BOD method. In the past years, several researchers have developed biosensors based on dissolved oxygen (DO) probes and immobilized cells for use as the biological recognition element [2]. Such systems generally give a good relationship between the output signal and BOD concentration, but may suffer from unstable operation [3]. 

A microbial fuel cell (MFC) is a device that uses microorganisms as catalysts to generate electricity from organic or inorganic fuels. It thus provides a potential approach for the generation of renewable energy or for powering of electronic sensors [3,4,5]. It can be operated with or without mediators by using an electrochemically active bacterium or a microbial consortium. Considering the operating convenience and cost, the mediatorless MFC is appropriate for developing a BOD biosensor [6]. The MFC biosensor is a promising method for applications in BOD measurements because it has broad substrate versatility, good operating stability, and high result reproducibility [5]. Although some studies related to MFC-type BOD biosensors have been presented, MFC biosensors inoculated with mixed cultures of unknown strains require continuous maintenance after construction of the MFC [7]. Requirement of another MFC biosensor with similar performance makes construction difficult because the composition of the mixed culture is unknown. This problem is probably where the greatest uncertainty in MFC design lies. Such uncertainty significantly limits the commercial application of MFC biosensors. Hence, Hsieh and Chung developed a MFC biosensor inoculated with known mixed cultures. They were able to use the established system to measure BOD concentrations in various wastewater samples in batch mode [7,8].

In a fixed MFC infrastructure, parameters affecting the MFC performance include fuel type, coexisting ions, electron acceptors, liquid retention time (LRT) in the anode, and gas retention time (GRT) in the cathode [9,10]. In earlier studies, a glucose–glutamate BOD standard solution has been used often to evaluate MFC performance in measuring BOD in wastewater samples. However, such studies focused on evaluating compounds such as carbohydrates, proteins, and lipids as BOD mode substrates are limited. Theoretically, a high BOD concentration leads to a high signal output. However, this is only true to a certain extent. Under anaerobic, anoxic, and microaerophilic conditions, microbes utilize organic substrates through different metabolic pathways, following Monod growth kinetics, and producing different electric outputs [11,12]. Hence, it is important to understand the effect of various fuels on the MFC performance in measuring BOD concentration in water samples [3].

Some ions such as Na^+^, K^+^, Ca^2+^, Mg^2+^, Cl^−^, and NH_4_^+^ exist at much higher concentrations than that of H^+^ in wastewater, thus potentially affecting the function of traditional ion-exchange membranes in MFCs [5,13]. Influent wastewater containing Fe^2+^, Mn^2+^, Zn^2+^, Cu^2+^, and Cr^6+^ ions may be toxic to microbes in the MFC and may thereby impair the MFC performance. Thus, evaluating the effects of coexisting ions on MFC operations is necessary. Although the MFC-type BOD biosensor has been shown to have long-term operational stability [8], the signal produced from MFC biosensor is significantly reduced when electron acceptors with high redox potential (oxygen, nitrate, nitrite, or sulfate) are present in the influent [14]. To measure accurately the BOD concentration in samples, acceptors in the influent should be removed from the influent wastewater. Azide, cyanide, and rotenone, known respiratory inhibitors, may reduce interference from electron acceptors when added to the influent [15]. 

In our previous research, a mediatorless MFC biosensor inoculated with a known bacterial mixture was developed for rapid measurement of BOD in “batch” mode. An operating procedure for the MFC biosensor that includes inoculation, immobilization, startup, and operation was established and tested [7]. In this study, a similar mediatorless MFC biosensor inoculated with a known bacterial mixture was constructed to measure BOD concentrations in various wastewater samples. The effects of important operating parameters on establishing reliable calibration curves between BOD concentration and output signals were evaluated. These parameters included influent fuel types, coexisting ions in the influent, electron acceptors in the influent, LRT in the anode, and GRT in the cathode. The amended method was successfully demonstrated. Our results could increase the commercial value of a MFC-type BOD biosensor.

## 2. Materials and Methods

### 2.1. MFC

The dual chamber mediatorless MFC constructed used a BOD biosensor, as previously described [7]. The surface area of the electrodes and the membrane used in the reactor were 0.0098 and 0.0072 m^2^, respectively. The MFC biosensor for BOD measurement was inoculated with *Thermincola carboxydiphila*, *Pseudomonas aeruginosa*, *Ochrobactrum intermedium*, *Shewanella frigidimarina*, *Citrobacter freundii*, and *Clostridium acetobutylicum*, which were isolated from the original MFC biosensor. All bacteria were cultured in Luria-Bertani (LB) medium until 1.0 OD_600_ and then the mixed cultures were added as inoculum. The cell number for *T. carboxydiphila*, *P. aeruginosa*, *O. intermedium*, *S. frigidimarina*, *C. freundii*, and *C. acetobutylicum* in the mixture was 8.2 × 10^8^, 3.5 × 10^9^, 2.0 × 10^9^, 6.8 × 10^8^, 2.5 × 10^9^, and 1.5 × 10^9^ CFU/ml, respectively. The immobilization process has been previously described [7]. A short description was as follows: 500 mL mixed cultures were put in a serum bottle and recycled by pump into the MFC at 10-day retention time under anaerobic condition. When the potential reached a steady state (after approximately 32 days of operation), the biofilm in the anode of the MFC was considered stable or mature.

The anode compartment was kept anoxic by purging with nitrogen gas, unless stated otherwise. The MFC influent was usually fed at 4 h LRT. Air was purged at 6 min GRT into the cathode compartment to supply O_2_ needed for the electrochemical reaction, unless stated otherwise. The catholyte contained 100 mM phosphate-buffered saline (PBS) and 100 mM NaCl solution. The MFCs were placed in a temperature-controlled chamber maintained at 35 °C. The external resistance of MFCs was set at 5000 Ω, obtained from the result of the previous polarization experiments [7]. The potential between the anode and cathode was continuously measured by using a multimeter (Model 2700, Keithley Instruments Inc., Cleveland, OH, USA).

### 2.2. Operating Properties of the MFC Biosensor

A 100 mg/L BOD solution containing 75.8 mg/L glucose and 75.8 mg/L glutamic acid (GGA) was used as the standard feed solution. Different liquid retention times in the anode chamber (1–6 h) and gas retention times in the cathode chamber (0–12 min) were adopted to examine the MFC performance. MFCs were fed with different types of substrates, each at 100 mg/L (carbohydrates: glucose, fructose, sucrose, lactose, and maltose; amino acids: glutamine, glycine, isoleucine, methionine, and phenylalanine; acids: acetic acid, propionic acid, and butyric acid; alcohols: ethanol and glycerol) and were operated at 4 h LRT and 6 min GRT to identify distinct metabolic pathways. To reduce the meddling of previous substrate residue, all data were collected after a time period of three times the hydraulic retention time. Additionally, selected substrates with 100 mg/L BOD_5_ were fed to the MFC to test the MFC performance under anaerobic conditions and to establish the calibration curve. A 100 mg/L GGA solution (as BOD_5_) containing various ions (Fe^2+^, Mn^2+^, Zn^2+^, Cu^2+^, Cr^6+^, or Cl^−^) at different concentrations were continuously introduced into the MFC to evaluate the effects of coexisting ions in the influent on BOD measurement using the MFC biosensor. The composition of the simulated wastewater containing 100 mg/L GGA solution (as BOD_5_) and electron acceptors (nitrate, nitrite, and sulfate) or metabolic inhibitors (rotenone, KCN, and NaN_3_) at various concentrations was continuously introduced to the MFC to understand their effects on BOD measurement using the MFC biosensor. BOD concentrations in three types of real wastewater (river water, seawater, and domestic wastewater) were measured by using the MFC biosensor and through the BOD_5_ standard method (five-day BOD test).

### 2.3. Analysis

Data from the multimeter were recorded by a personal computer through a data acquisition system (Testpoint, Capital Equipment Co., Richmond, VA, USA). The potential difference (voltage) and current were recorded under various operating conditions to evaluate the MFC performance. The measured voltage was converted to current according to the relationship, voltage = current × resistance. Current density is the electric current per unit total surface area of the anode. Biochemical oxygen demand analysis adopted the standard BOD method 5210 B. All experiments were conducted by using three separate MFCs. All sample analyses were carried out in triplicate, and mean values were reported.

## 3. Results and Discussion

### 3.1. Effect of Retention Time on MFC Performance

**Figure 1:**
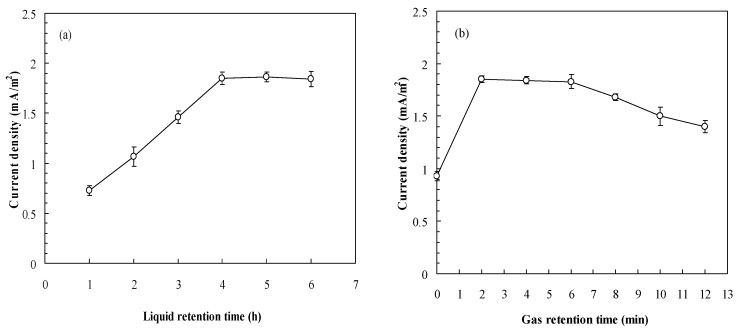
Effects of (**a**) Liquid retention time (GRT: 6 min); (**b**) Gas retention time (LRT: 4 h) on the MFC performance (GGA concentration: 100 mg/L as BOD_5_).

During continuous operation, liquid retention time in the system often determines the degradation efficiency of pollutants. For a MFC system, the degradation efficiency of pollutants may affect electricity generation. Generally, a low LRT means a high organic loading to the reactor, which should increase the voltage. However, a low LRT often results in the insufficient contact between substrates and microbes in the anode compartment, which may led to the decrease of the voltage. Thus, to obtain the optimized LRT is decisive. Figure 1a indicates that the current density of the MFC increases as LRTs increase, leveling off when the LRT is higher than 4 h. In a MFC system, oxygen is the electron acceptor in the cathode. Hence, the supply of sufficient oxygen favors electricity generation and enhances the signal output from the MFC. Figure 1b indicates the relationship between the current density of the MFC and GRT in the cathode. The results indicate that the optimal GRT range in the MFC cathode was 2–6 min. With too short a retention time (GRT: 0 min) or with insufficient oxygen (GRT: 8–12 min), the current generation of MFC is reduced. Thus, the LRT and GRT for MFC operation were respectively set at 4 h and 6 min in subsequent experiments.

### 3.2. Effect of Substrates on MFC Performance

Theoretically, a high BOD concentration in the influent leads to a higher voltage generated by the MFC; however, this is only true to a certain extent. Since microbes in the MFC anode utilize organic substrates through various metabolic pathways, electrical outputs may vary even at the same concentration [12]. Gil *et al.* showed that microbes in their developed MFC appeared to utilize glucose or acetate better [16]; thus, they were often used in the MFC as model fuels.

**Figure 2:**
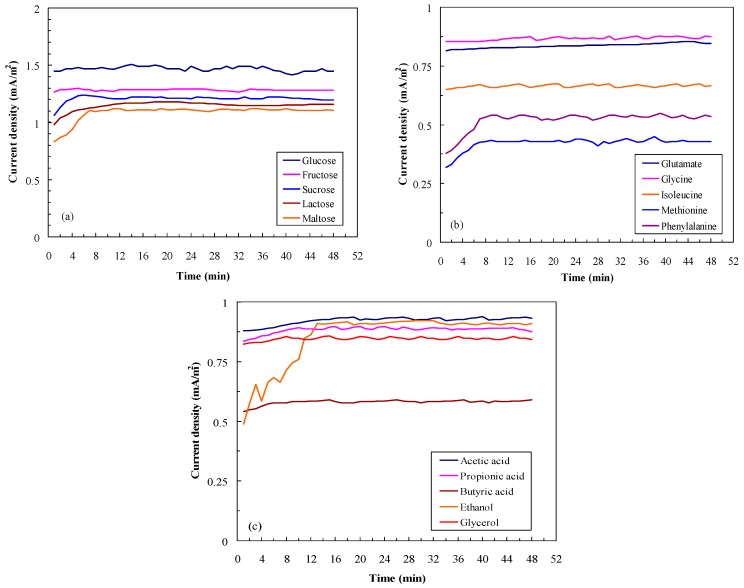
Effects of different substrates on the MFC performance. (**a**) Carbohydrates; (**b**) Amino acids; (**c**) Acids and alcohols (substrate concentration: 100 mg/L; LRT: 4 h; GRT: 6 min; n = 3).

Figure 2 shows the effects of different substrates at a concentration of 100 mg/L on the current density of the MFC in continuous feeding mode. Results indicate that monosaccharides and disaccharides were better substrates for electricity generation in the MFC than were amino acids, organic acids, or alcohols. The current density of 0.9 mA/m^2^ was thus used as a baseline. An output higher than 0.9 mA/m^2^ by the MFC is due to carbohydrate degradation, while lower current generation is due to degradation of other compounds (Figure 2). For carbohydrates, the current generation from the MFC fed with monosaccharides was higher than that of the MFC fed with disaccharides. Additionally, a stable current density was observed after 1–10 min (Figure 2a). The electricity generated from the MFC depended on the structures of the various amino acids. Among the tested amino acids, methionine and phenylalanine were relatively poorly degraded; therefore, they produced only 0.4–0.5 mA/m^2^ stable current density (Figure 2b). Figure 2c indicates that ethanol was the substrate that was the most difficult metabolize; hence, a stable current density was observed only after 15 min. In the following experiments, the BOD measurements were conducted after an average of 5 min of operation. 

**Figure 3:**
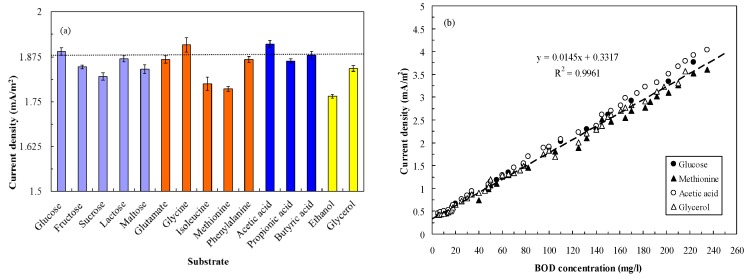
(**a**) Effects of different substrates on the MFC performance (substrate concentration: 100 mg/L as BOD_5_; LRT: 4 h; GRT: 6 min); (**b**) Relationship between BOD_5_ concentration and voltage output of the MFC biosensor (substrate concentration: 5–235 mg/L as BOD_5_; LRT: 4 h; GRT: 6 min).

To establish the calibration curve between the signal output and BOD_5_ concentration, the substrate concentration should be converted to the BOD_5_ concentration through the BOD standard method 5210 B. Figure 3a indicates the current density of the MFC during continuous feeding of the selected organic compounds at 100 mg/L BOD_5_ concentration. The results indicate the variation of the current generation by the MFC due to the degradation of different organic compounds, even at the same BOD concentration. A MFC fed with 100 mg/L GGA solution generated 1.880 ± 0.012 mA/m^2^, but MFCs fed with glycine (1.908 ± 0.021 mA/m^2^) and acetic acid (1.910 ± 0.013 mA/m^2^) generated more electricity (*p* < 0.05). When MFCs were fed with sucrose, isoleucine, methionine, and ethanol as fuels, lower current generation was observed. 

This decrease may be attributed to the degradation of organic compounds through different metabolic pathways [17]. To offset the bias of the current generation from different fuels, different BOD_5_ concentrations of glucose, methionine, acetic acid, and glycerol were used to establish the calibration curve. Figure 3b illustrates the relationship between the BOD_5_ concentration and the current density of the MFC biosensor in continuous feeding mode. A good linear relationship could be observed at BOD_5_ concentrations ranging from 5 to 235 mg/L. The regression equation for the BOD concentration and the current density of the MFC biosensor was determined to be *y* = 0.0145*x* + 0.3317 (r^2^ = 0.9961). In their studies, Chang *et al.* [4], who used a dual-chamber MFC, and Kumlanghan *et al.* [18], who used a large MFC (1.1 L), also observed linear relationships when inlet BOD concentrations were in the range of 20–100 mg/L and 1–25 g/L, respectively. In comparison with the previous systems, our continuous monitoring system showed some competitive advantages because of our lower limit of detection. The regression equation was thus applied in calculating the BOD_5_ concentration of different water samples.

### 3.3. Effect of Coexisting Ions on MFC Performance

The use of ion-exchange membranes to separate the anode and cathode in the MFC is well known [3]. The flow of electrons must be accompanied by an equal flow of protons or hydroxide ions through the ion-exchange membrane. Thus, electron-transfer efficiency is an important factor in the control of MFC performance [19]. Actual water samples such as river water, seawater, and domestic wastewater contain various ions, and the presence of these ions in the samples may interfere with electron transfer in the MFC and may affect the activity of microbes in the anode [20]. 

**Figure 4:**
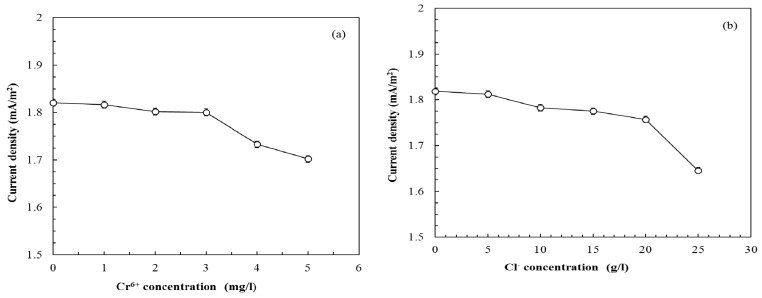
Effects of (**a**) Cr^6+^ concentration; (**b**) Cl^−^ concentration on the MFC performance (GGA concentration: 100 mg/L as BOD_5_; LRT: 4 h; GRT: 6 min).

The difference in current generation between the control and the anolyte containing the metal ions Fe^2+^, Mn^2+^, Zn^2+^, and Cu^2+^ was insignificant (*p* > 0.05) when the ion concentration in the anode was below 5 mg/L. However, when Cr^6+^ concentrations in the anolyte were higher than 3 mg/L, the reduction of current density was significant and the responses had relative values of 93%–95% of that of the control (Figure 4a). Bachate *et al.* demonstrated that the toxicity of Cr^6+^ to microbes is due to its higher solubility in water and favorable permeability through biological membranes [21]. In addition, Cr^6+^ can act as an electron acceptor in the anode under anaerobic conditions. These reasons may explain why the current density of the MFC dropped. The effects of chloride ions on the responses of the MFC biosensor current generation were also investigated. The average Cl^−^ concentration in seawater was approximately 20 g/L [22]. Figure 4(b) shows that the responses of the MFC biosensor are insignificantly influenced by the increasing Cl^−^ concentration (0–20 g/L). Thus, these results suggest that the MFC biosensor has great potential for BOD measurements on different water bodies, including seawater. This advantage may be attributed to the characteristics of the bacterial mixtures inoculated in the anode, which effectively degrade complex organic compounds and survive in toxic conditions [7].

### 3.4. Effects of Metabolic Inhibitors

Previous research has shown that the signal from MFC-type BOD biosensors is significantly reduced when electron acceptors with high redox potential are present in the influent [14]. Wastewater samples from rivers, domestic wastewater, seawater, or industrial wastewater generally contain oxygen gas (O_2_), nitrate (NO_3_^-^), nitrite (NO_2_^−^), and even sulfate SO_4_^2–^ [23]. Thus, electron acceptors in the influent to the MFC biosensor should be removed to enable accurate measurement of the BOD concentration in these samples. 

**Figure 5:**
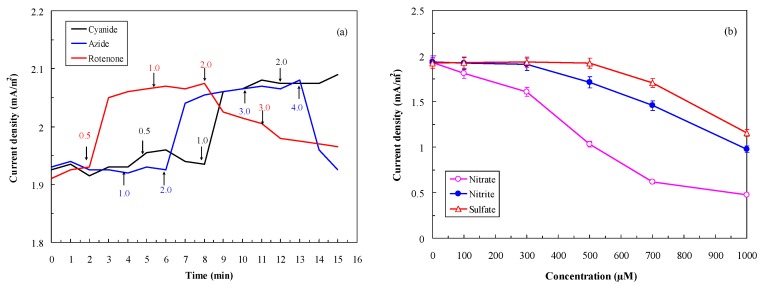
Effect of (**a**) Respiratory inhibitors (cyanide, azide, rotenone); (**b**) Electron acceptors (nitrate, nitrite, and sulfate) in the influent on the MFC performance (GGA concentration: 100 mg/L as BOD_5_; LRT: 4 h; GRT: 6 min; n = 3).

Figure 5a shows the effect of respiratory inhibitors (cyanide, azide, and rotenone) on preventing interference from oxygen gas. In the experiment, the tested influent contained 100 mg/l GGA BOD_5_ solution with 4.5 mg/L DO (without nitrogen purge) or 100 mg/L GGA BOD_5_ solution under anaerobic conditions (data not shown). Results indicate that the anaerobic control generated 2.061 ± 0.018 mA/m^2^ of current density (data not shown) and that the current density of the tested influent decreased to an average of 1.922 ± 0.024 mA/m^2^. With continuous addition of respiratory inhibitors at different concentrations to the MFC, the current density of the tested influent gradually increased and leveled off at the current density of the anaerobic control (2.061 ± 0.018 mA/m^2^). The appropriate respiratory inhibitors were rotenone (0.5 mg/L), cyanide (1.0 mg/L), and azide (2.0 mg/L). Figure 5b shows the effect of electron acceptors (NO_3_^−^, NO_2_^−^, SO_4_^2–^) in the MFC influent on the performance. In the experiment, the tested influent contained 100 mg/L GGA BOD_5_, 4.5 mg/L DO, and different concentrations of electron acceptors. Results indicate that nitrate in the influent reduced most strongly the current generation by the MFC, followed by nitrite and sulfate. The concentrations of NO_3_^−^, NO_2_^−^, and SO_4_^2–^ in inhibiting current generation were 100, 500, and 700 μM, respectively. 

**Figure 6:**
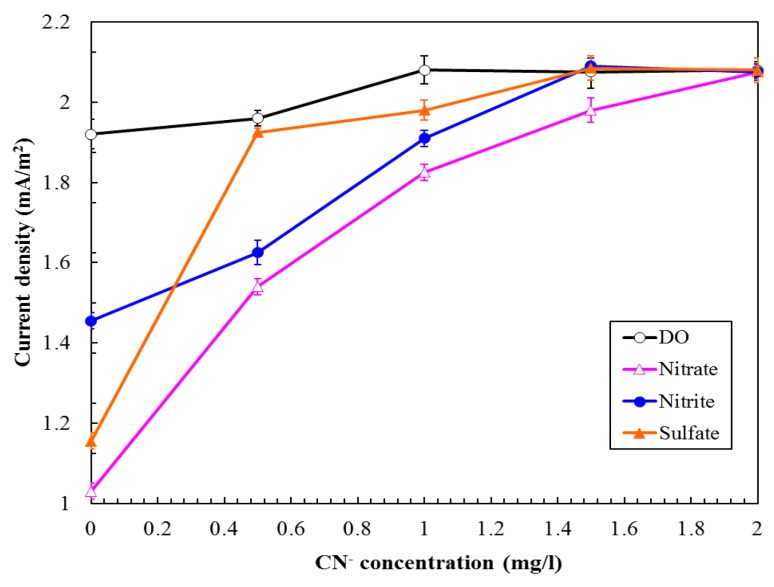
Effect of cyanide concentration in the influent on the MFC performance. (GGA concentration: 100 mg/L as BOD_5_; oxygen gas concentration: 4.5 mg/L; nitrate concentration: 500 μM; nitrite concentration: 700 μM; sulfate concentration: 1000 μM; LRT: 4 h; GRT: 6 min).

Different concentrations of cyanide (CN^−^) were continuously added to the MFC biosensor to examine the interference of coexisting electron acceptors and to evaluate the MFC performance. In the experiment, the tested influent contained 100 mg/L GGA BOD_5_, 4.5 mg/L DO (without nitrogen purge), and different electron acceptors. NO_3_^−^, NO_2_^−^, and SO_4_^2–^ were fed at 500, 700, and 1000 μM, respectively. Figure 6 shows the improvement in the current generation by the MFC with electron acceptors due to different CN^−^ concentrations. Results indicate that the current from the MFC increased with CN^−^ concentration. The current density returned to the level (*i.e.*, 2.061 mA/m^2^) under anaerobic conditions (control) upon addition of CN^−^ to a 1–2 mg/L final concentration. Azide, cyanide, or rotenone inhibit nitrate reductase, nitrite reductase, or NADH dehydrogenase [17,24,25], thus improving the electron transfer from the anode to the cathode in the MFC. Therefore, the MFC biosensor can measure BOD_5_ concentrations in various water samples even when they contain electron acceptors.

### 3.5. Continuous BOD Measurement on Wastewater Using the MFC Biosensor

Different water bodies usually contain organics, trace metals, toxic compounds, and electron acceptors. These coexisting compounds may affect BOD measurements by the MFC biosensor. Thus, it is crucial to evaluate the feasibility of using a MFC biosensor for continuous BOD measurements on different actual wastewater samples. In this study, wastewater samples were fed to the MFC biosensor at 4 h LRT, 6 min GRT, and 2 mg/L CN^−^ was simultaneously added to reduce the interference of coexisting electron acceptors.

Table 1 summarizes the results of BOD concentrations in actual wastewater samples either continuously measured by using the MFC biosensor or measured through the BOD_5_ standard method. MFC biosensor measurements of BOD concentration in river water and seawater had low deviations (−3.33 to 2.00%) relative to those from the BOD_5_ standard method. Furthermore, BOD concentration measurements on domestic wastewater using the MFC biosensor (<210 mg/L) were accurate and had low deviations (−3.08 to 1.06%) relative to those obtained through the BOD_5_ standard method. However, deviations were relatively high (6.88%–7.58%) when domestic wastewater contained 320–580 mg/L BOD. Kim *et al.* [8] treated artificial wastewater by using a mediatorless MFC in batch mode, and Nakamura *et al.* [22] treated real river water by using a double-mediator MFC system in batch mode. Similarly, Kumlanghan *et al.* [26] treated food wastewater by using a large MFC in continuous mode, Zhang and Angelidaki [27] treated contaminated groundwater by using a SUMFC sensor in batch mode, and Hsieh and Chung [7] treated wastewater by using a mediatorless MFC in batch mode. Deviations for these studies are, respectively, 0.65%–2.6%, 14.5%–20%, −12%–−15%, 6%–16%, and 2.5%–3.6% relative to that of the BOD_5_ standard method [7,8,22,26,27]. Findings from the present study suggest that the MFC biosensor has some competitive advantages in continuous BOD measurement because of its low deviation relative to that of the BOD_5_ standard method.

**Table 1:** BOD measurements by the MFC biosensor and the BOD standard method.

	River Water			Seawater		Domestic Wastewater				
	A*	B	C	A	B	A	B	C	D	E
BOD_5_ (mg/L)	18 ± 1.6	5±0.6	26 ± 1.8	15 ± 0.9	6.5 ± 0.4	210 ± 10.2	65 ± 2.8	25 ± 1.8	320 ± 13.8	580 ± 15.6
MFC biosensor	17.8 ± 1.2	5.1 ± 0.3	25.2 ± 1.2	14.5 ± 0.6	6.3 ± 0.2	206 ± 5.8	63 ± 1.9	24.6 ± 1.2	342 ± 8.5	624 ± 12.1
Deviation (%)	−1.11	2.00	−3.08	−3.33	−3.08	−1.9	−3.08	−1.6	6.88	7.58

* Different letters mean real water sample obtained from different sources.

## 4. Conclusions

A MFC biosensor for fast, accurate, and continuous BOD measurements was constructed by using an inoculated known bacterial mixture as the biological sensing element. The 4 h LRT in the anode and the 6 min GRT in the cathode of MFC enabled maximal generation of electricity. Various organic compounds in the MFC influent resulted in generation of different amounts of electricity due to different metabolic pathways, even at the same BOD concentration in the influent. To offset the bias of the current generation derived from different fuels, a linear relationship between the BOD_5_ concentration and the current density of the MFC biosensor in continuous mode was established and tested. Coexisting ions (Fe^2+^, Mn^2+^, Zn^2+^, and Cu^2+^) in the influent had an insignificant effect on the MFC performance when the ion concentration in the anode was below 5 mg/L. Adding cyanide without any pretreatment could significantly alleviate the effect of electron acceptors in the influent on the MFC performance. The MFC biosensor is advantageous in that its deviation is low relative to that of the standard five-day BOD test, even during application in continuous BOD measurements on wastewater samples. Thus, the MFC biosensor has great potential use in an online instrument for accurate BOD measurements for water bodies.
